# Malaysian Parents’ Willingness to Vaccinate Their Children against COVID-19 Infection and Their Perception of mRNA COVID-19 Vaccines

**DOI:** 10.3390/vaccines10111790

**Published:** 2022-10-25

**Authors:** Li-Ping Wong, Hai-Yen Lee, Haridah Alias, Sazaly AbuBakar

**Affiliations:** 1 Centre for Epidemiology and Evidence-Based Practice, Department of Social and Preventive Medicine, Faculty of Medicine, University of Malaya, Kuala Lumpur 50603, Malaysia; 2 Tropical Infectious Diseases Research and Educational Centre (TIDREC), University of Malaya, Kuala Lumpur 50603, Malaysia

**Keywords:** childhood vaccination, COVID-19 vaccine, mRNA vaccine, parent attitude, vaccine hesitancy

## Abstract

Little was known about Malaysian parental attitudes, beliefs, and intentions surrounding coronavirus disease 2019 (COVID-19) vaccines for children when the National COVID-19 Immunization Program for Children (PICKids) was launched in February 2021. A cross-sectional online survey-based study was carried out from 15 March 2022 to 23 July 2022 on Malaysian parents/guardians of children between ages 5 and below 12 years old. A total of 15.7% reported being extremely willing, and 38.9% were somewhat willing to vaccinate children with a COVID-19 vaccine. Perceived low susceptibility to COVID-19 infection showed the greatest significant impact on vaccine acceptance (OR 35.46, 95% CI 15.26–82.40). Parents with a lower level of concern have a higher willingness for vaccination (OR 1.25, 95% CI 0.90–1.75). Of the parents that knew of the mRNA vaccine, 46.6% reported that they prefer their children to be vaccinated with conventional vaccines over mRNA vaccines. Poor knowledge about mRNA vaccines, lack of confidence in the mRNA technology, fear of unknown side effects, and perception that the mRNA vaccines contain microchips were significantly associated with a higher level of concern about their children receiving an mRNA vaccine. Public education campaigns to promote COVID-19 vaccination for children warrant addressing the concerns and knowledge deficits among vaccine-hesitant parents.

## 1. Introduction

The outbreak of the coronavirus disease 2019 (COVID-19) was initially detected in Wuhan, China in December 2009. The World Health Organization (WHO) on 11 March 2020, declared the COVID-19 outbreak a global pandemic [1]. The COVID-19 pandemic has spread rapidly across the globe. In Malaysia, the first case of COVID-19 infection was confirmed on 25 January 2022. The global effort to develop effective vaccines against the coronavirus disease (COVID-19) has brought new hope to fight the outbreak and minimize its negative impact. Since the worldwide roll-out of COVID-19 vaccines, COVID-19 vaccination has been shown to contribute to reducing severe illness from COVID-19 deaths and reducing the transmission of COVID-19 [2]. The worldwide economy has begun to recover, with schools and businesses reopening. Widespread COVID-19 vaccination uptake is essential for achieving sufficient immunization coverage to end the global pandemic. However, as of September 2022, only approximately 5.35 billion people worldwide have received a COVID-19 vaccine, which is equal to an estimated 69.7% of the world population [3]. This denotes that there is still a significant portion of the global population who are unable to get access to the vaccines, deferring or declining to get vaccinated for COVID-19. COVID-19 vaccine hesitancy is a major barrier to reaching a sufficient number of people to achieve herd immunity to SARS-CoV-2 [4], therefore, with vaccination efforts underway for adults, it is important to recognize the importance of COVID-19 vaccination in children, and that many adults who are parents may be hesitant to immunize their children.

The US Food and Drug Administration (FDA) expanded the emergency use authorization for the COVID-19 vaccine to include adolescents 12 through to 15 years on 10 May 2021 [5]. Subsequently, on 29 October 2021, the FDA authorized the use of Pfizer (NY, USA) and BioNTech’s COVID-19 vaccine (Mainz, Germany) for children ages 5 to 11 [6]. Despite the recognition that COVID-19 vaccines have received authorization from stringent regulatory authorities for children and adolescents [7], many remain unwilling to vaccinate their children against COVID-19. Parental vaccine hesitancy has serious consequences for children and is also a major barrier to achieving herd immunity across populations. Some studies reported that parental vaccine hesitancy is more pronounced with the COVID-19 vaccines than with other routine childhood vaccines [8,9].

Vaccine hesitancy is influenced by a wide range of individual-level determinants [10], hence it should be the target in combating parental vaccine hesitancy. The personal perceived threat of COVID-19 has been one of the most commonly studied personal health belief factors affecting COVID-19 vaccine hesitancy [11]. Various studies showed that COVID-19 vaccine hesitancy in adults was associated with a low perceived risk of getting infected [11,12]. Similarly, a low perceived risk of infection was associated with parental hesitancy toward child COVID-19 vaccination [13].

With the COVID-19 vaccine being newly developed, in most of the recent studies, the primary concerns expressed by vaccine-hesitant parents have pertained to vaccine safety, effectiveness, and the risk of their children developing adverse effects from the vaccine [14,15,16,17]. A study in Malaysia similarly reported that parents were primarily concerned about the COVID-19 vaccine being a new vaccine [18]. Not only did the rapid development and the newness of the COVID-19 vaccines yield worries about vaccine safety, but the concern about the use of the messenger RNA (mRNA) vaccine platform was also reported in many international [19,20,21] as well as Malaysian studies [22,23]. The public may lack confidence in vaccines developed using mRNA technology over the traditional inactivated virus and recombinant protein platforms, and the mRNA vaccines have been subjected to many conspiracy theories since they were launched [24]. Misleading information about RNA-based vaccines has resulted in a high degree of vaccine hesitancy [19,20,21].

Malaysia started its COVID-19 vaccination program for adults in February 2021. The National COVID-19 Immunization Program for Children (PICKids) was launched in February 2022, where children aged 5 to under 12 years are offered the Pfizer-BioNTech COVID-19 mRNA vaccine [25]. Therefore, this study aimed to investigate parental hesitancy toward the child’s COVID-19 vaccination and its influencing factors. Additionally, to the best of our knowledge, parental acceptance or refusal of the mRNA vaccine offered under the PICKids program has never been investigated. In the event that the COVID-19 vaccine available is not their choice, parents may refuse, delay, or are hesitant to vaccinate their children. Hence, this study also determines the knowledge, perception, and acceptance regarding their child getting an mRNA COVID-19 vaccine. Children have been adversely impacted by school closure, social isolation, and confinement, which have had negative impacts on children’s mental health and well-being. With the COVID-19 vaccine roll-out underway, getting children vaccinated enables children to return to normal activities.

## 2. Materials and Methods

### 2.1. Study Participants and Survey Design

A nationwide cross-sectional study was conducted from 20 March 2022 to 23 July 2022, through the dissemination of an online survey. The inclusion criteria were that the respondents were Malaysians ≥ 18 years and parents/guardians of at least one child of age 5 to under 12 years who have not received COVID-19 vaccination. The calculated sample size was 385, based on a normal approximation of binomial distribution with a finite population correction applied, assuming an observer proportion of respondents selecting a specific response option of 50%, a 95% confidence level, and a margin of error of 5%. The sample size was multiplied by the predicted design effect of two to account for the use of convenience sampling and an online survey. Therefore, the minimum sample size for this study was 770 (385 × 2) participants. A convenient sampling method was used. The survey link on Google Forms was sent out to the general public. Respondents who completed the survey received a note encouraging them to disseminate the survey link to everyone in their contact list.

### 2.2. Measures

The demographic characteristics of parents were first determined. Subsequently, the survey questionnaire (Supplementary Materials) was divided into two sections. The first section assessed (1) willingness to vaccinate their children against COVID-19; (2) perception of children’s risk of acquiring COVID-19; and (3) concern about COVID-19 vaccination for children. The second section assessed parents’ knowledge and attitudes on mRNA vaccines, with questions consisting of (1) mRNA vaccine acceptance; (2) knowledge of mRNA vaccines; (3) perception of mRNA vaccines.

#### 2.2.1. Parents’ Demographics

The demographic questions were related to age, gender, ethnicity, and average monthly household income.

#### 2.2.2. Willingness to Vaccinate Their Children

Participants were asked to rate their level of willingness to vaccinate their children against COVID-19. The response options were *extremely willing, somewhat willing, undecided, somewhat not willing,* and *not willing.*

#### 2.2.3. Perception of Risk of Acquiring COVID-19

COVID-19 risk perception is a single-item question that queried parents’ perceptions of their children’s susceptibility to COVID-19 infection, with the response options of *strongly disagree, disagree, agree,* and *strongly agree.*

#### 2.2.4. Concern about COVID-19 Vaccination for Children

A 4-item question assessing parents’ concern regarding adverse side effects following vaccination, unknown long-term side effects, and risk of COVID-19 infection and death following vaccination, with the response options of *not at all concerned, slightly concerned, moderately concerned,* and *extremely concerned.*

#### 2.2.5. mRNA Vaccines Acceptance

Parents were first asked about their preference for the type of COVID-19 vaccine for their children. The optional answers were *mRNA COVID-19 vaccine, conventional COVID-19 vaccine,* and *no preference.* Parents were also asked to rate their level of concern about their children getting an mRNA vaccine. The response options were *not at all concerned, slightly concerned, moderately concerned,* and *extremely concerned.*

#### 2.2.6. Knowledge of mRNA Vaccines

A total of four questions were used to measure the level of knowledge of the mRNA vaccines. The questions assess whether participants know the difference between mRNA and conventional vaccines and if they know how mRNA vaccines work. The answer choices given to the respondents were *true*, *false,* and *don’t know*. The responses were rated as 1 for correct responses and 0 for incorrect or “don’t know” responses. The total score ranges from 0 to 4, with higher scores indicating a higher level of knowledge.

#### 2.2.7. Attitudes on mRNA Vaccines

Participants were asked five questions in the assessment of attitudes toward mRNA vaccines. The first question asked whether they have confidence in the mRNA technology; second, whether they worry about unknown side effects of the mRNA vaccine; third, whether they viewed conventional vaccines as safer; fourth, whether mRNA vaccines generate a stronger immune response; and five, whether mRNA vaccines may contain a microchip. The response options of *strongly disagree, disagree, agree,* and *strongly agree* were given.

All the questions were content validated. Content validation involved consulting with content experts and academicians to evaluate whether the items in the questionnaire were clear and represented the intended construct. The content experts were also asked to evaluate whether the items covered the study objectives and if there were missing components. A forced answering option was implemented where all questions had to be answered before the participant could move on to the next page to avoid incomplete responses, hence there are no missing data. Data checking and cleaning were carried out before analyses to ensure valid and reliable responses. Straightlining and duplicate responses were checked and removed.

### 2.3. Statistical Analysis

Descriptive analyses were performed to examine the distribution of all variables of interest, including normality, means, and frequencies. Knowledge item scores were totaled. The score for the knowledge items was not normally distributed, so the median and interquartile range (IQR) are used to describe the score. Cronbach’s alpha was calculated for the vaccine confidence scale to assess reliability in terms of internal consistency. The knowledge scale had good internal consistency, with Cronbach’s alpha of 0.975. Binary logistic regression and multivariate logistic regression were used to explore: (1) the factors affecting the willingness to vaccinate children against COVID-19 (1 = *slightly/moderately/ extremely concerned*; 0 = *not at all concerned*); (2) the factors affecting the level of concern about vaccinating children with a COVID-19 mRNA vaccine (1 = *slightly/moderately/ extremely concerned*; 0 = *not at all concerned*). The variables with *p* < 0.05 in the binary logistic regression analyses were entered into the multivariate logistic regression model. The odds ratios (ORs) with 95% confidence intervals (CIs) were computed for each independent variable. The model fit was assessed using the Hosmer–Lemeshow goodness-of-fit test. The Hosmer–Lemeshow statistic indicated a poor fit if the significance value is < 0.05 [26]. All analyses were also conducted using SPSS version 22.0 (SPSS Inc., Chicago, IL, USA).

### 2.4. Ethical Considerations

This study was implemented according to the principles of the Declaration of Helsinki and granted ethical approval by the Universiti Malaya Research Ethics Committee (UM.TNC2/UMREC–1795). The survey did not collect any identifying information from participants. Respondents were also informed that their participation was voluntary. To consent to participate, participants were required to click ‘Yes, I consented to participate in this study’.

## 3. Results

### 3.1. Baseline Characteristics of the Population

A total of 1003 parents participated in this study. Baseline characteristics of the study population are shown in the first and second columns of Table 1. Females accounted for 66.7% and 45.3% were aged 35 to 59 years old. Nearly two-thirds (64.6%) of the parents reported an average monthly household income of MYR5001–MYR10,000.

### 3.2. Intention for Vaccination and Influencing Factors on Acceptance of COVID-19 Vaccine

**Figure 1:**
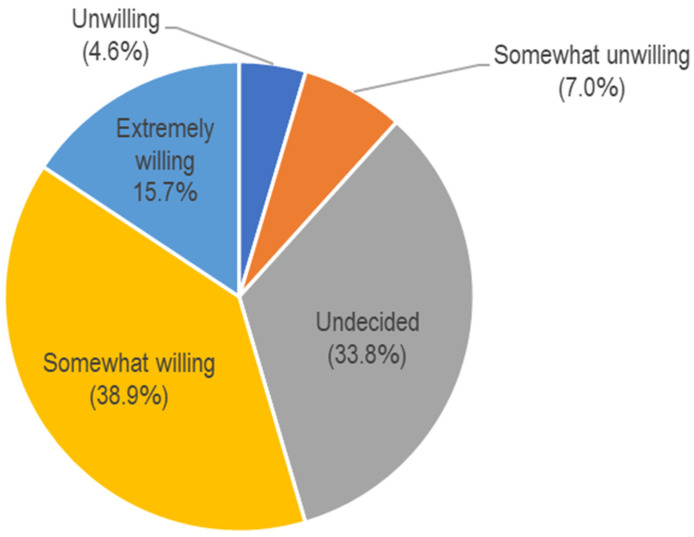
Proportion of responses of willingness to vaccinate children with a COVID-19 vaccine (N = 1003).

In the parents’ responses to the willingness to vaccinate children, 15.7% reported being extremely willing, 38.9% were somewhat willing, and 33.8% reported that they were undecided (Figure 1). Factors influencing willingness to vaccinate children are presented in Table 1. By demographics, older age-group parents were associated with a higher willingness to vaccinate children against COVID-19 (OR 2.63, 95% CI 1.66–4.16). By ethnic groups, Indian (OR 0.39, 95% CI 0.25–0.59) express a lower willingness to vaccinate their children against COVID-19 than Malay parents. A small proportion of parents (9.6%) agreed that the COVID-19 vaccine for children is not needed as children are not as susceptible to COVID-19 infection. Perceived low susceptibility to COVID-19 infection showed the greatest significant impact on vaccine acceptance in the multivariate model (OR 35.46, 95% CI 15.26–82.40). In response to concerns about vaccinating their children, a high proportion reported extreme concern about the risk of death (76.6%), potential unknown long-term side effects (74.9%), severe adverse effects (71.3%), and risk of COVID-19 infection (68.5%). Only the concern about potential unknown long-term side effects was found to be significantly associated with willingness to vaccinate their children in the univariate analysis. In the multivariate analysis, parents who express a lower level of concern have a higher willingness for vaccination (OR 1.25, 95% CI 0.90–1.75). Nonetheless, the association was not significant in the multivariate model.

### 3.3. mRNA Vaccines Acceptance and Attitudes

**Figure 2:**
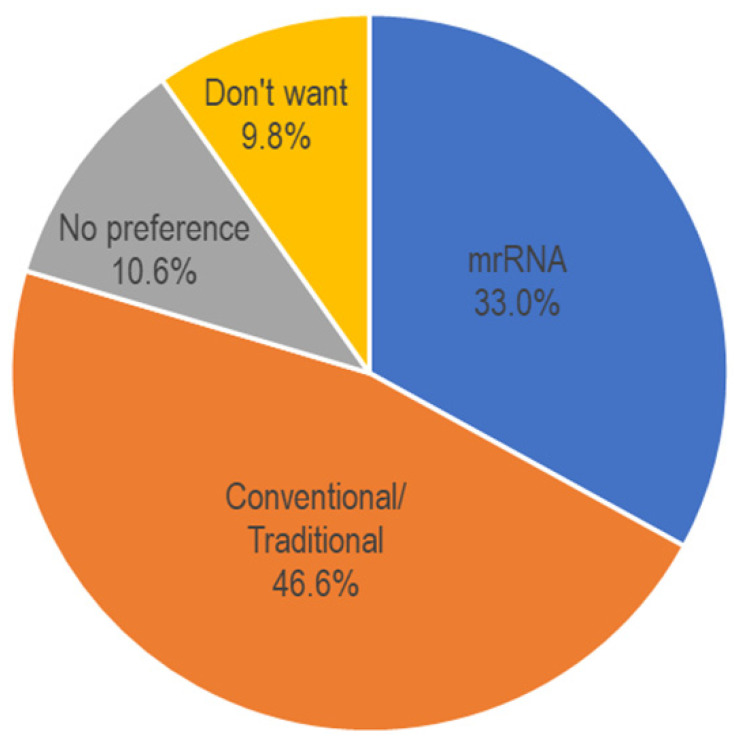
Preference of a COVID-19 vaccine for child (N = 917).

**Figure 3:**
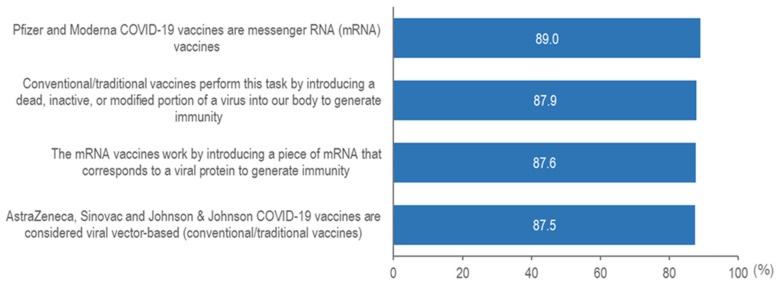
Proportion of correct responses of knowledge related to COVID-19 mRNA vaccines (N = 917).

A total of 86 (8.6%) parents reported not knowing what an mRNA vaccine is. Of the 917 parents that answered the second section of the questionnaire, the majority of study participants reported that they’d prefer their children to be vaccinated with conventional or traditional vaccines over mRNA vaccines (46.6%), whereas 33.0% of parents preferred mRNA vaccines and 10.6% reported no preferences (Figure 2). Figure 3 shows the proportion of correct responses to knowledge questions. The majority of study participants reported correct responses for all the items. The average knowledge score for participants was 3.3 (SD = 1.5, range 0–4), representing a good level of knowledge on mRNA vaccines.

The findings regarding attitudes toward mRNA vaccines are shown in the first and second columns of Table 2. Over half of parents (56.3%) reported being worried about the unknown side effects of the mRNA vaccines. Nearly half (49.0%) believed the conventional COVID-19 vaccines are safer than mRNA vaccines. Over one-third were confident of the mRNA technology (38.5%) and viewed that the mRNA vaccines generate a stronger immune response than conventional vaccines. A small proportion (4.8%) responded in agreement with the statement that the mRNA vaccines may contain microchips.

In response to concerns about their children getting an mRNA vaccine, 40.0% (n = 399) were not at all concerned about their children receiving an mRNA vaccine. Table 2 shows the factors associated with the level of concern about mRNA vaccines. Parents from an average household income of MYR5000 and below reported a significantly higher level of concern than those with an income above MYR10000 (OR 11.65, 95% CI 3.84–35.33). A lower knowledge score (score 0–3) was associated with a higher level of concern about mRNA vaccines for their children than those with maximum knowledge of a score of 4 (OR 4.49, 95% CI 2.07–9.74). With regard to attitude factors influencing the level of concern about mRNA vaccines, the multivariable model shows that low confidence in the mRNA technology, fear of unknown side effects, and the perception that the mRNA vaccines contain microchips were significantly associated with a higher level of concern about their children receiving an mRNA vaccine.

## 4. Discussion

This study assessed parents’ willingness to vaccinate their children against COVID-19, and their acceptance and perception of mRNA COVID-19 vaccines approximately one month after the COVID-19 Immunization Program for children aged 5 to under 12 years old commenced in Malaysia. To date, there has been limited empirical evidence on parental COVID-19 vaccine acceptance, particularly in Malaysia. The present study is one of the first to be conducted in Malaysia to understand the parents’ willingness and attitudes toward vaccinating young children against COVID-19 and provides important insights into addressing challenges faced in the future introduction of new vaccination plans for children.

In the present study, only 15.7% of parents reported being extremely willing and 38.9% were somewhat willing to vaccinate their children against COVID-19. A higher willingness to vaccinate children against COVID-19 was found in a similar study conducted approximately 6 months after the introduction of COVID-19 vaccination for children in Malaysia, in which the study found that 73.6% of parents reported a willingness to immunize their children [18]. This may imply that parents were hesitant to vaccinate their children with the COVID-19 vaccine in the initial introduction of COVID-19 vaccination for children and acceptance has increased over time. The National Pharmaceutical Regulatory Agency (NPRA) has not received any reports of serious side effects resulting from COVID-19 vaccines given under the children’s COVID-19 vaccination program (PICKids) for children aged five to under 12 years in Malaysia; only several non-serious adverse effects from the coronavirus vaccine have been reported [27], this perhaps has led to parents being more confident in the COVID-19 vaccination program for their children as time passes. As of October 2022, 86% of the adult population has received at least one dose of a COVID-19 vaccine and 84% has received the booster dose [28]. By contrast, only 43% of children between 5 and 11 years old in Malaysia are fully vaccinated against COVID-19 [28]. This implies that despite a considerable proportion of Malaysian parents being vaccinated against COVID-19, they have a higher hesitancy to have their children vaccinated. During the pandemic of infectious diseases, prompt uptake of recommended vaccination is important to decrease the disease burden and successfully control the pandemic. Efforts to enhance vaccine confidence in parents during the introduction of a new vaccination are important to prevent a delay in vaccinations.

The present study also provides evidence that low perception of susceptibility of children getting COVID-19 is associated with higher parental hesitancy toward child COVID-19 vaccination, similarly to that found in another study [29]. Although empirical evidence is showing that children below 10 years old exhibited lower susceptibility to COVID-19 compared to adults [30], nevertheless, it should be pointed out that COVID-19 infections leading to serious illness, hospitalization, or death are also common in children, especially in those with previous comorbidities [31]. A recently published epidemiological study of pediatric COVID-19 infections found that the rate of severe/critical COVID-19 disease was 0.8% to 5.3% in Singapore, Malaysia, Japan, and China, although higher rates were reported in India (16%), Indonesia (37.5%), and Pakistan (78%) [32]. Hence, public health messages must be tailored to imparting messages that increase parental appraisal of the threat of the coronavirus disease to children.

The current study identified vaccine safety and potential side effects as top concerns about COVID-19 vaccines for children; additionally, unknown long-term side effects are powerful factors influencing parents’ willingness to vaccinate their children against COVID-19. The COVID-19 vaccines undergo rigorous testing in clinical trials to meet high safety standards before their roll-out to the general population. It is well recognized that convincing parents of the COVID-19 vaccination for their children have been more challenging than addressing COVID-19 vaccine hesitancy in adults. To date, safety data on children were much more limited than on adults, but most children experience mild side effects after being vaccinated and cases of deaths recorded are rare [33]. Plenty of studies prove that the benefits of vaccination outweigh any risks or concerns, except in rare cases [34]. Public health education messages for parents should focus on informing parents that current scientific evidence supports the safety of the COVID-19 vaccines for children. Parents should also be made aware of the public health benefits of the COVID-19 vaccines and that the benefits of vaccination outweigh the side effects.

Our study also found that a considerable proportion of parents prefer conventional vaccines over mRNA vaccines. Similarly, a considerable proportion also expressed concern over their child receiving an mRNA vaccine. Restriction on vaccine choice is one of the major contributors to COVID-19 vaccine hesitancy [22]. With PICKids, during the early phase of the vaccination program, the health authorities were offering Pfizer Inc.’s COVID-19 vaccine for children aged between 5 and 11 years old. Findings imply the need to build confidence in mRNA COVID-19 vaccines along with the introduction of the COVID-19 vaccination program, particularly if the vaccination program only offers mRNA vaccines. This study also found that, after adjustment for demographic factors, a lack of knowledge about the mRNA vaccines was associated with a higher level of concern about mRNA vaccines for their children. Furthermore, low confidence in mRNA technology, fear of unknown side effects, and a perception that the mRNA vaccines contain microchips were significantly associated with a higher level of concern about their children receiving an mRNA vaccine. Our findings provide insight into parent-targeted intervention messages to enhance mRNA vaccine uptake. The current state-of-art of mRNA vaccines is an effective, fast, and cost-effective response to emerging health crises [35], hence this should be made known to parents. Parents should also be informed that mRNA vaccines have promising safety profiles, with only mild or moderate adverse events occurring in clinical trials [36]. Currently, positive safety and efficacy data are abundant concerning mRNA vaccines, along with a proven path to regulatory approval [36]. Hence, providing empirical evidence-based information to enhance parents’ confidence in the mRNA COVID-19 vaccine is essential. There is also a need to halt anti-vaccine controversies claiming the COVID-19 vaccine contains tracking microchips that are vigorously circulating on social media.

Some limitations of our study should be noted. The first limitation is the cross-sectional design: we identified associations but could not infer cause and effect. It is also important to note that online survey methods may lead to a biased response from people who have good Internet literacy and access as well as those who are more educated. Additionally, convenient sampling may result in a sample that was lacking in the representativeness of the general population. Therefore, the results should be interpreted with caution. Despite these limitations, the respondents of the study presented a broad spectrum of demographic backgrounds, which contributes tremendously to the understanding of parents’ responses to the introduction of COVID-19 vaccination for their young children in Malaysia.

## 5. Conclusions

In this study, slightly over half of the parents were extremely willing or somewhat willing to vaccinate their children of age 5 to under 12 years against COVID-19 approximately one month after the COVID-19 Immunization Programme was initiated. High perceived susceptibility among children to COVID-19 infection had a significant effect on parents’ willingness to vaccinate their children. Therefore, future health education for the COVID-19 vaccine should focus on tackling misbeliefs about low-risk perceptions that led to an underestimation of the threat of COVID-19 infection in young children. Concerns about the vaccine’s safety and potential side effects of COVID-19 vaccines in children were paramount and fear of unknown long-term side effects is a powerful factor influencing parents’ willingness to vaccinate their children against COVID-19, hence interventions to promote COVID-19 vaccination for children may be most effective by addressing these concerns.

This study also revealed a considerable proportion of parents were concerned about mRNA vaccines and preferred conventional vaccines over mRNA vaccines. A lack of knowledge about the mRNA vaccines was the prime reason for a higher level of concern about mRNA vaccines for their children. In particular, low confidence in the mRNA technology, fear of unknown side effects, and a perception that the mRNA vaccines contain microchips were parents’ main concerns. Personalized health education initiatives based on these knowledge deficits may reduce parents’ concerns about the COVID-19 vaccination for children. Promoting the uptake of mRNA COVID-19 vaccines requires an understanding of the concerns found in this study. Our findings are enormously important to inform effective communications with parents who are prone to vaccine refusal or hesitation.

## Figures and Tables

**Table 1:** Demographic characteristics and factors associated with willingness to vaccinate children against COVID-19 (N = 1003).

		Univariable Analysis			Multivariable Analysis
		Willingness to Vaccinate Children against COVID-19			Willingness to Vaccinate Children against COVID-19
	N (%)	Not Willing at all/ Somewhat Unwilling/ Undecided (n = 458)	Somewhat Willing/ Definitely Willing (n = 547)	*p*-Value	Somewhat Willing/ Definitely Willing vs. Not Willing at all/ Somewhat Unwilling/ Undecided
Socio-demographic characteristics					
Age group (years)					
23–34	295 (29.4)	154 (51.5)	143 (48.5)	*p* < 0.001	Reference
35–39	454 (45.3)	217 (47.8)	237 (52.2)		1.36 (0.97–1.90)
40–72	254 (25.3)	87 (34.3)	167 (65.7)		2.63 (1.66–4.16) ***
Gender					
Male	336 (33.5)	134 (39.9)	202 (60.1)	0.013	1.32 (0.98–1.77)
Female	667 (66.7)	322 (48.3)	345 (51.7)		Reference
Ethnicity					
Malay	198 (19.7)	88 (44.4)	110 (55.6)	*p* < 0.001	Reference
Chinese	195 (19.4)	71 (36.4)	124 (63.6)		0.72 (0.44–1.17)
Indian	481 (48.0)	250 (52.0)	231 (48.0)		0.39 (0.25–0.59) ***
Others	129 (12.9)	47 (36.4)	82 (63.6)		0.77 (0.45–1.33)
Average monthly household income (MYR)					
5000 and below	103 (10.3)	63 (61.2)	40 (38.8)	*p* < 0.001	Reference
5001–10,000	648 (64.6)	299 (46.1)	349 (53.9)		0.56 (0.29–1.10)
More than 10,000	252 (25.1)	94 (37.3)	158 (62.7)		0.50 (0.23–0.59)
Perceived susceptibility					
The COVID-19 vaccine for children is not needed as children are not as susceptible to COVID-19 infection co					
Strongly agree/Agree	96 (9.6)	87 (90.6)	9 (9.4)	*p* < 0.001	Reference
Disagree/ Strongly disagree	907 (90.4)	369 (40.7)	538 (59.3)		35.46 (15.26–82.40) ***
Concerns over the COVID-19 vaccination for child					
Severe adverse effects after COVID-19 vaccination					
Not at all concerned/ Slightly concerned/ Moderately concerned	288 (28.7)	118 (41.0)	170 (59.0)	0.080	
Extremely concern	715 (71.3)	338 (47.3)	377 (52.7)		
Unknown long-term side effects					
Not at all concerned/ Slightly concerned/ Moderately concerned	252 (25.1)	98 (38.9)	154 (61.1)	0.016	1.25 (0.90–1.75)
Extremely concern	751 (74.9)	358 (47.7)	393 (52.3)		Reference
Risk of COVID-19 infection					
Not at all concerned/ Slightly concerned/ Moderately concerned	316 (31.5)	136 (43.0)	180 (57.0)	0.306	
Extremely concern	687 (68.5)	320 (46.6)	367 (53.4)		
Risk of death					
Not at all concerned/ Slightly concerned/ Moderately concerned	235 (23.4)	101 (43.0)	134 (57.0)	0.410	
Extremely concern	768 (76.6)	355 (46.2)	413 (53.8)		

*** *p* < 0.001, Hosmer–Lemeshow test, chi-square: 10.299, *p*-value: 0.245; Nagelkerke R^2^: 0.207.

**Table 2:** Factors associated with level of concern of mRNA vaccines (N = 917).

		Univariable Analysis			Multivariable Analysis
		Level of Concern about Vaccinating Children with a COVID-19 mRNA Vaccine			Level of Concern of Vaccinating Children with a COVID-19 mRNA Vaccine
	N (%)	Not at all Concerned (n = 366)	Slightly/ Moderately/ Extremely Concerned (n = 551)	*p*-Value	Slightly/ Moderately/ Extremely Concerned vs. Not at all Concerned
Socio demographic characteristics					
Age group (years)					
23–34	268 (29.2)	105 (39.2)	163 (60.8)	0.665	
35–39	431 (47.0)	176 (40.8)	255 (59.2)		
40–72	218 (23.8)	85 (39.0)	133 (61.0)		
Gender					
Male	296 (32.3)	116 (39.2)	180 (60.8)	0.773	
Female	621 (67.7)	250 (40.3)	371 (59.7)		
Ethnicity					
Malay	165 (18.0)	53 (32.1)	112 (67.9)	0.099	
Chinese	176 (19.2)	73 (41.5)	103 (58.5)		
Indian	461 (50.3)	187 (40.6)	274 (59.4)		
Others	115 (12.5)	53 (46.1)	62 (53.9)		
Average monthly household income (MYR)					
5000 and below	80 (8.7)	7 (8.8)	73 (91.3)	*p* < 0.001	11.65 (3.84–35.33) ***
5001–10,000	594 (64.8)	254 (42.8)	340 (57.2)		1.00 (0.52–1.93)
More than 10,000	243 (26.5)	105 (43.2)	138 (56.8)		Reference
Knowledge of mRNA COVID-19 vaccine					
Total knowledge score					
Score 0–3	136 (14.8)	36 (26.5)	100 (73.5)	*p* < 0.001	4.49 (2.07–9.74) ***
Score 4	781 (85.2)	330 (42.3)	451 (57.7)		Reference
Attitudes towards COVID-19 mRNA vaccine					
I have confidence in the new and advanced technological approach used in the development of the COVID-19 mRNA vaccine					
Strongly agree/Agree	353 (38.5)	277 (78.5)	76 (21.5)	*p* < 0.001	Reference
Disagree/ Strongly disagree	428 (46.7)	12 (2.8)	416 (97.2)		3.40 (0.74–15.68)
Don’t know	136 (14.8)	77 (56.6)	59 (43.4)		0.28 (0.09–0.84) *
I am worried there might be unknown side effects of the mRNA vaccines that will show up months or years later					
Strongly agree/Agree	516 (56.3)	22 (4.3)	494 (95.7)	*p* < 0.001	23.54 (7.87–70.35) ***
Disagree/ Strongly disagree	283 (30.9)	258 (91.2)	25 (8.8)		0.97 (0.29–3.25)
Don’t know	118 (12.9)	86 (72.9)	32 (27.1)		Reference
The conventional/ traditional COVID-19 vaccines are safer than the COVID-19 mRNA vaccines					
Strongly agree/Agree	449 (49.0)	21 (4.7)	428 (95.3)	*p* < 0.001	2.24 (0.69–7.31)
Disagree/Strongly disagree	274 (29.9)	238 (86.9)	36 (13.1)		0.54 (0.17–1.73)
Don’t know	194 (21.2)	107 (55.2)	87 (44.8)		Reference
The COVID-19 mRNA vaccines generate a stronger immune response than the conventional/traditional vaccines					
Strongly agree/Agree	322 (35.1)	266 (82.6)	56 (17.4)	*p* < 0.001	Reference
Disagree/ Strongly disagree	413 (45.0)	15 (3.6)	398 (96.4)		1.20 (0.26–5.64)
Don’t know	182 (19.8)	85 (46.7)	97 (53.3)		1.57 (0.47–5.29)
The COVID-19 mRNA vaccines may contain microchip					
Strongly agree/Agree	44 (4.8)	1 (2.3)	43 (97.7)	*p* < 0.001	2.31 (0.19–27.54)
Disagree/Strongly disagree	437 (47.7)	281 (64.3)	156 (35.7)		0.35 (0.16–0.74) **
Don’t know	436 (47.5)	84 (19.3)	352 (80.7)		Reference

* *p* < 0.05, ** *p* < 0.01, *** *p* < 0.001. Hosmer–Lemeshow test, chi-square: 35.322, *p*-value: *p* < 0.001; Nagelkerke R^2^: 0.821.

## Data Availability

The datasets used and/or analyzed during the current study are available from the corresponding author on reasonable request.

## Supplementary Materials

The following supporting information can be downloaded at: https://www.mdpi.com/article/10.3390/vaccines10111790/s1, Survey Questionnaire.
