# Supplementary material for: Malaysian Parents’ Willingness to Vaccinate Their Children against COVID-19 Infection and Their Perception of mRNA COVID-19 Vaccines
Source: Vaccines (Basel). 2022 Oct 25;10(11):1790. doi: 10.3390/vaccines10111790 (PMC9693578; doi:10.3390/vaccines10111790)
Supplement: Supplementary file 1 [file vaccines-10-01790-s001.zip › vaccines-1994424-supplementary.pdf]

PARENTS' PERCEPTION ABOUT THE COVID-19 VACCINES AND WILLINGNESS TO VACCINATE THEIR CHILDREN AGAINST COVID-19 INFECTION

Section A

GENERAL INFORMATION

1	Age	______________________ years old
2	Gender	[1] Male<br>[2] Female
3	Ethnicity	[1] Malay<br>[2] Chinese<br>[3] Indian<br>[4] Bumiputera Sabah/Sarawak<br>[5] Others, please specify:<br>______________________
4	Monthly average household income (MYR)	______________________<br>[1] MYR 1,000 and below<br>[2] MYR 1,001 to 2,000<br>[3] MYR 2,001 to 3,000<br>[4] MYR 3,001 to 4,000<br>[5] MYR 4,001 to 5,000<br>[6] MYR 5,001 to 6,000<br>[7] MYR 6,001 to 7,000<br>[8] MYR 7,001 to 8,000<br>[9] MYR 8,001 to 9,000<br>[10] MYR 9,001 to 10,000<br>[11] More than MYR 10,000

Section B

Willingness to vaccinate your child with COVID-19 vaccine

1	What is your willingness to vaccinate your child against COVID-19 infection?	[1] Extremely Willing<br>[2] Somewhat willing<br>[3] Undecided<br>[4] Somewhat not willing<br>[5] Not willing

Section C

Perception of the risk of acquiring COVID-19

1	The COVID-19 vaccine for children is not needed as children are not as susceptible to COVID-19 infection compared to adults.	[1] Strongly disagree<br>[2] Disagree<br>[3] Agree<br>[4] Strongly agree

Section D

Concerns about COVID-19 vaccination for children

The following is the list of concerns over COVID-19 vaccines for children. Please rate your level of concern/worry.

1	Severe adverse effects after COVID-19 vaccination	[1] Not at all concerned<br>[2] Slightly concerned<br>[3] Moderately concerned<br>[4] Extremely concerned
2	Unknown long-term side effects that may show up months or years later.	[1] Not at all concerned<br>[2] Slightly concerned<br>[3] Moderately concerned<br>[4] Extremely concerned
3	Children may get COVID-19 from the vaccines	[1] Not at all concerned<br>[2] Slightly concerned<br>[3] Moderately concerned<br>[4] Extremely concerned
4	Risk of death following COVID-19 vaccination	[1] Not at all concerned<br>[2] Slightly concerned<br>[3] Moderately concerned<br>[4] Extremely concerned

Section E

mRNA vaccines Acceptance

1	Preference of type of COVID-19 vaccine for your child	[1] mRNA covid-19 vaccine<br>[2] Conventional COVID-19 vaccine<br>[3] No preference
2	How concerned (or worried) are you about your child getting an mRNA COVID-19 vaccine?	[1] Not at all concerned<br>[2] Slightly concerned<br>[3] Moderately concerned<br>[4] Extremely concerned<br>[5] I don't know what is mRNA vaccine

Section F

Knowledge of mRNA vaccines

1	AstraZeneca, Sinovac and Johnson &Johnson COVID-19 vaccinees are considered viral vector-based (conventiontional/ traditional vaccines)	[1] True	[2] False	[3] Don't know/Not sure
2	Pfizer and Moderna COVID-19 vaccines are messenger RNA vaccines	[1] True	[2] False	[3] Don't know/Not sure
3	Conventional/traditional vaccines perform this task by introducing a dead, inactive, or modified portion of a virus into our body to generate immunity.	[1] True	[2] False	[3] Don't know/Not sure
4	The mRNA vaccines work by introducing a piece of mRNA that corresponds to a viral protein to generate immunity.	[1] True	[2] False	[3] Don't know/Not sure

Section G

Perception on mRNA vaccines

1	I have confidence in the new and advanced technological approach used in the development of the COVID-19 mRNA vaccine	[1] Strongly disagree<br>[2] Disagree<br>[3] Agree<br>[4] Strongly agree<br>[5] Don't know/no opinion
2	I am worried there might be unknown side effects of the mRNA vaccines that will show up months or years later.	[1] Strongly disagree<br>[2] Disagree<br>[3] Agree<br>[4] Strongly agree<br>[5] Don't know/no opinion
3	The conventional/traditional COVID-19 vaccines are safer than the COVID-19 mRNA vaccines	[1] Strongly disagree<br>[2] Disagree<br>[3] Agree<br>[4] Strongly agree<br>[5] Don't know/no opinion
4	The COVID-19 mRNA vaccines generate a stronger immune response than the conventional/traditional vaccines	[1] Strongly disagree<br>[2] Disagree<br>[3] Agree<br>[4] Strongly agree<br>[5] Don't know/no opinion
5	The COVID-19 mRNA vaccines may contain microchips	[1] Strongly disagree<br>[2] Disagree<br>[3] Agree<br>[4] Strongly agree<br>[5] Don't know/no opinion
